# Differential antiviral effects and immune responses in nasal and airway organoid during RSV infection: implications for interferon therapy

**DOI:** 10.3389/fimmu.2026.1754206

**Published:** 2026-02-18

**Authors:** Linmei Wang, Lina Chen, Lin Yang, Yanan Hu, Danli Lu, You Duan, Li Qiu, Yan Li, Rui Zhang, Hanmin Liu, Wenhao Yang

**Affiliations:** 1 Department of Pediatric Pulmonology, West China Second University Hospital, Sichuan University, Chengdu, China; 2 Key Laboratory of Birth Defects and Related Diseases of Women and Children (Sichuan University), Ministry of Education, Chengdu, China; 3 National Health Commission (NHC) Key Laboratory of Chronobiology (Sichuan University), Chengdu, China; 4 The Joint Laboratory for Lung Development and Related Diseases of West China Second University Hospital, Sichuan University and School of Life Sciences of Fudan University, West China Institute of Women and Children’s Health, West China Second University Hospital, Sichuan University, Chengdu, China; 5 Sichuan Birth Defects Clinical Research Center, West China Second University Hospital, Sichuan University, Chengdu, China; 6 Department of Pediatric Pulmonology and Immunology, West China Second University Hospital (WCSUH)-Tianfu·Sichuan Provincial Children's Hospital, Sichuan University, Meishan, China; 7 Xizang Region Child Development Clinical Medical Research Center, Lhasa, China

**Keywords:** child, immunity, interferon, organoids, respiratory syncytial virus

## Abstract

**Introduction:**

Respiratory syncytial virus (RSV) is a leading cause of lower respiratory tract infections in infants and young children, and it constitutes a significant risk factor for the development of bronchiolitis and subsequent childhood asthma. The severity of the disease is notably higher in infants compared to adults, underscoring the urgent need for effective therapeutic interventions.

**Methods:**

In our study, we utilized pediatric nasal and airway epithelial organoids to demonstrate that both type I and type III interferons (IFNs) markedly reduce viral load and downregulate key inflammatory mediators, including IL-6, CXCL8, IL-1α, and TNF, during RSV infection. Through transcriptome Sequencing and multiplex cytokine profiling of 46 immune mediators, we observed a more robust immune response in the nasal epithelium compared to the airway epithelium.

**Results:**

Notably, IFN-λ1 was most effective in suppressing inflammation in the nasal epithelium, whereas IFN-β did not exacerbate inflammatory responses in the airway epithelium.

**Discussion:**

These findings provide novel insights for optimizing clinical IFN therapy, particularly in terms of selecting the appropriate interferon type, delivery site, and dosing strategy.

## 1 Introduction

Respiratory syncytial virus (RSV) is the leading pathogen causing lower respiratory tract infections in children under 5 years old worldwide, particularly in infants (1). It contributes significantly to morbidity and mortality rates, with the most severe disease burden observed in low- and middle-income countries (2). Severe RSV infection causes age-specific clinical syndromes, such as sepsis-like illness in neonates, bronchiolitis in infants, and pneumonia in toddlers (3). Furthermore, RSV infection in early childhood is closely associated with significant long-term sequelae, including recurrent wheezing, asthma, and increased susceptibility to lower respiratory infections (4, 5), representing a major public health challenge. Potential mechanisms by which early-life RSV infection may lead to asthma include alterations in epithelial cell barrier function, mucosal and systemic innate responses, adaptive humoral and cellular immune responses, and the airway microbiome (6).

Three classes of interventions are under development for infant RSV protection: extended half-life monoclonal antibodies (mAbs), maternal vaccines, and pediatric vaccines (7). Pediatric vaccines are not yet available. In contrast, the approved maternal RSVpreF vaccine works by immunizing pregnant individuals and transferring antibodies across the placenta, providing newborns with early protection. It has shown high efficacy, preventing 81.8% and 69.4% of severe RSV-associated lower respiratory tract illness within 90 and 180 days after birth, respectively (8). The passive immunization with mAbs—palivizumab, nirsevimab, and clesrovimab—constitutes the primary preventive approach for infants (9–11). Nirsevimab has been approved in multiple regions worldwide, providing protection throughout the entire respiratory syncytial virus (RSV) season, with significant efficacy in safeguarding high-risk infants such as preterm neonates and those with congenital heart disease. It is crucial to note that these are prophylactic agents and are not effective against active RSV infection.

The innate immune response serves as the host’s first line of defense against viral infections, with the interferon (IFN) system playing a pivotal role in restricting viral replication during early infection. Sequencing analyses of nasopharyngeal swabs from COVID-19 patients have indicated that an early interferon response signature in the nasal mucosa correlates with reduced disease severity, whereas diminished expression of interferon-stimulated genes (ISGs) is associated with poorer outcomes (12). In infants and young children, the immaturity of the immune system, combined with viral strategies that actively suppress endogenous interferon production, increases susceptibility to viral infections (13, 14). Therefore, timely administration of exogenous interferon may be particularly vital during the early stages of infection.

The interferon system is broadly categorized into three types: type I (IFN-I), type II (IFN-II), and type III (IFN-III) (15). Type I interferons comprise multiple subtypes, with IFN-α and IFN-β being the most prevalent. While nearly all nucleated cells can produce type I interferons upon infection, plasmacytoid dendritic cells (pDCs) are their primary source. IFN-γ, the sole type II interferon, is mainly secreted by T lymphocytes, natural killer (NK) cells, and antigen-presenting cells such as monocytes, macrophages, and dendritic cells (16). Type III interferons, a more recently identified group, include IFN-λ1 (IL-29), IFN-λ2 (IL-28A), IFN-λ3 (IL-28B), and IFN-λ4 (17). IFN-λ is predominantly produced by epithelial cells, and its receptor is also primarily expressed on epithelial cells. Functionally similar to type I interferons, type III interferons serve as key guardians of barrier integrity at mucosal surfaces by promoting pathogen clearance and suppressing excessive inflammation to maintain mucosal homeostasis (18).

Interestingly, viral load may not be the only factor that drives disease. In some cases, the host response to RSV may be described as overexuberant, inappropriate, or dysregulated (19). For example, some studies of children with severe or fatal bronchiolitis describe lung inflammation with a pronounced monocytic, T cell, and neutrophilic infiltrate (20) and an abundance of inflammatory mediators in the airway fluids (21, 22), and many animal studies of RSV disease highlight the role of the excessive host response in causing disease (23). Current approved treatments for hospitalized RSV patients include bronchodilators, corticosteroids, and antiviral agents (24–26). Among these therapeutic strategies, interferon holds a central position due to its dual mechanisms of broad-spectrum antiviral activity and immunomodulation. According to the Expert consensus on the rational application of interferon alpha in pediatrics (27) and a supporting clinical study (28), nebulized interferon has been utilized in the treatment of RSV, though its use remains off-label.

Anatomically, both the nasal cavity and airways are lined with pseudostratified epithelium composed of basal cells, ciliated cells, secretory cells, and goblet cells (29). Whole-exome sequencing revealed that over 99% of single nucleotides are shared between human nasal epithelial organoids(NO) and homolog airway epithelial organoids(AO), with 95% overlap in their RNA transcriptomes (30). Upon RSV infection, the cytokine response in nasal epithelium coordinates early innate and subsequent adaptive immune responses, thereby playing a crucial role in controlling viral replication and preventing spread to the lower respiratory tract. The absence of suitable preclinical models has significantly hindered research into RSV pathogenesis and treatment development in infants. To address this, we utilized three-dimensional primary NO and AO derived from infant donors to evaluate the dual role of interferon in suppressing RSV viral load and mitigating the associated cytokine response. This study specifically aims to identify the optimal interferon type and dosage that effectively balances antiviral activity with immunomodulatory effects.

## 2 Materials and methods

### 2.1 Ethics approval statement

Throughout the study, we adhered to the principles of the Declaration of Helsinki. The cohort comprised three children (a 2-year-old boy, a 4-year-old girl, and a 7-year-old girl). Participants in our study presented with recurrent respiratory infections suggestive of primary ciliary dyskinesia (PCD), necessitating bronchoscopy-guided transmission electron microscopy (TEM) examination for definitive diagnosis. Ultimately, PCD was excluded due to negative TEM and whole-exome sequencing (WES) results, along with normal ciliary beat frequency (CBF). PCD exclusion was based on diagnostic criteria outlined in the European Respiratory Society guidelines (31). The study protocol was approved by the Ethics Review Committee of West China Second University Hospital, Sichuan University (KL118). All participating family members provided written informed consent.

### 2.2 Organoid culture

The detailed procedures for obtaining NO and AO from bronchoscopy and nasal swab samples were previously described by our group (30). Briefly, nasal swabs and tracheal biopsy specimens from the same patient were preserved in DMEM and immediately transported to the laboratory on ice at 4°C. Tracheal biopsy samples were digested in digestion buffer at 37°C with shaking for 1 hour and agitated with a pipette tip every 30 minutes. Digestion was terminated with FBS, filtered through a 40-μm filter, and the cell pellet was obtained by centrifugation at 300g for 3 minutes. Nasal swabs were repeatedly rinsed with a 1-mL pipette tip to obtain sufficient cells, filtered through a 100-μm filter, and the cell pellet was obtained by centrifugation at 300g for 3 minutes. Both cell pellets were washed twice with wash buffer, centrifuged at 300g for 3 minutes, and supernatants discarded. 30 µl of Matrigel was added to each well of a 24-well plate and incubated at 37°C for 15 minutes to allow for solidification. Then, 500 µl of complete medium was added to each well, and the plate was incubated at 37°C with 5% CO_2_;. The medium was changed every 4 days. Once the organoids reached a diameter of 200 µm, they were passaged and expanded. The sample was resuspended in wash medium and centrifuged at 200 g for 3 minutes. TrypLE 1× (Gibco, A1217701) was added, followed by incubation at 37°C for 10 minutes. The mixture was filtered through a 40 µm filter and centrifuged at 300g for 3 minutes to obtain the cell pellet. The pellet was washed twice with wash solution (centrifugation at 300 g for 3 minutes each time), the supernatant was discarded, and the cells were reseeded into Matrigel at a density of 5,000 cells per well. At each passage, a designated number of organoids were cryopreserved in cryopreservation solution (NCM Biotech, C40100) and stored in liquid nitrogen for future use. The WASH buffer consisted of premium DMEM/F12 containing 1× GlutaMAX, 10 mM HEPES, and antibiotics. The digestion solution comprised 1 mL of WASH buffer supplemented with 400 U/mL collagenase I (Sigma, St. Louis, MO, USA; 9001–12−1), 0.25 mg/mL trypsin E (Sigma, P5147), 10 μM Y27632 (Selleck, Houston, TX, USA; S6390), and 10 U/mL DNase I (Sigma, 10104159001). The complete medium was based on WASH buffer and contained 1× B27 (Gibco, Grand Island, New York, NY, USA, 0080085SA), 5 mM nicotinamide (Sigma, N0636), 1.25 mM N-acetylcysteine (Sigma, A0737), 500 nM SB202190 (Selleck, S1077), 500 nM A-8301 (Selleck, S8301), 500 ng/mL R-spondin1 (R&D, Minneapolis, MN, USA, 4645), 25 ng/mL recombinant human FGF7 (PeproTech, NJ, USA, 450-61), 100 ng/mL recombinant human FGF10, 100 ng/mL recombinant human Noggin (R&D, 6057), and 5 μM Y27632 (CST, Botton, MA, USA, 13624).

### 2.3 RNA extraction

Total RNA was isolated from the organoids via an RNAprep Pure Micro Kit (Tiangen, Beijing, China, CA, DP420). cDNA was subsequently generated with a Transcriptor First Strand cDNA Synthesis Kit (Roche, Basel, Switzerland, 04897030001) following the manufacturer’s instructions.

### 2.4 Immunofluorescence

Harvested organoids were fixed in 4% paraformaldehyde for 24 hours, dehydrated, embedded in paraffin, and sectioned into 5μm-thick slices. Sections underwent antigen retrieval by boiling in EDTA solution for 30 minutes, followed by blocking in 5% BSA buffer for 60 minutes to minimize non-specific staining. Primary antibodies targeting ACE-tubulin (Santa Cruz, Santa Cruz, CA, USA, sc-23950), P63 (Abcam, Cambridge, Waltham, MA, USA, ab124762), SCGB1A1 (Santa Cruz, CA, USA, sc-365992), MUC5AC (Abcam, Cambridge, Waltham, MA, USA, ab198294) were applied to the sections, followed by incubation at 4°C for 24 hours. After washing with PBS in three 10-minute intervals, sections were exposed to secondary antibodies labeled with Alexa Fluor 488 (Invitrogen, A11001) and Alexa Fluor 594 (Invitrogen, A11012) at room temperature for 1 hour. Cells were counterstained with Hoechst 33,342 (Invitrogen, Life Technologies, Carlsbad, CA, USA, H3570). The organoids were then washed three more times and sealed with anti-fluorescence quenching sealant (YEASEN, Shanghai, China, CA, 36307ES08). Finally, images were captured using a laser scanning confocal microscope (Olympus, Waltham, MA, USA).

### 2.5 Viral infection

RSV A2 (ATCC, Manassas, VA, USA VR-1540) obtained from Chongqing Medical University was cultured in HEp-2 cells (ATCC) and its viral titer was determined according to a standardized protocol (32). Infection with RSV virus was performed in a BSL-2 laboratory. Organoids with hollow lumens, grown in Matrigel domes, were gently dislodged by pipetting, digested with TrypLE for 1 min, washed twice, and centrifuged at 100g for 3 min. supernatant discarded. 2 μl virus (1 × 10^8^ pfu/ml) was added into 200μl organoid medium with sheared organoids in a 48-well plate (final virus concentration: 1 × 10^6^ pfu/mL, MOI = 10) (33). After 6 hours of incubation at 37°C and 5% CO_2_;, infected organoids were washed twice with wash medium, then resuspended in Matrigel and re-seeded as described above. We added an equal volume of DPBS or interferon (1, 10, or 100 ng/mL) to the complete medium, as used in prior 2D experiments (34, 35). Plates were incubated at 37°C for 48 hours, after which supernatants and samples were collected for subsequent experiments. Based on previous cell line studies and our initial findings, the 48-hour time point was determined to be optimal for observing immune response and cellular activation (30, 36). The following human recombinant protein were exogenously added: Human IFN-α2a (PeproTech^®^, Cat# 300-02AA-20UG), Human IFN-β (PeproTech^®^, Cat#300-02BC-20UG), Human IFN-γ (PeproTech^®^, Cat# 300-02-100UG), Human IL-29 (IFN-λ1) (PeproTech^®^, Cat# 300-02L-20UG).

### 2.6 Droplet digital PCR

The droplet digital PCR procedures adhered to the manufacturer’s guidelines for the QX200 Droplet Digital PCR System utilizing the supermix for probes (no dUTP) from Bio-Rad, Hercules, CA, USA. The final reaction volume was 20 µL and comprised 10 µL of 2× supermix for probes (no dUTP) from Bio-Rad; 2 µL of cDNA derived from 100 ng of RNA from the target sample; 0.5 µL each of the RSV-N-F, RSV-N-R, and RSV-N-P primers; and 6.5 µL of nuclease-free water. The 20-µL mixture was subsequently transformed into droplets via a QX200 droplet generator from Bio-Rad. The droplet-partitioned samples were subsequently transferred to a 96-well plate, sealed, and subjected to cycling in a T100 Thermal Cycler (Bio-Rad) according to the following cycling protocol: an initial step at 95°C for 10 min for DNA polymerase activation, followed by 40 cycles of denaturation at 94°C for 30 s and annealing at 58°C for 1 min, and a final step at 98°C for 10 min, with a subsequent hold at 4°C. The cycled 96-well plate was then moved to a QX200 reader (Bio-Rad) for reading in the FAM and HEX channels.

### 2.7 Transcriptome sequencing

Total RNA was extracted from the tissue using TRIzol^®^ Reagent according the manufacturer’s instructions. Then RNA quality was determined by 5300 Bioanalyser (Agilent) and quantified using the ND-2000 (NanoDrop Technologies). RNA purification, reverse transcription, library construction and sequencing were performed at Shanghai Majorbio Bio-pharm Biotechnology Co., Ltd. (Shanghai, China) according to the manufacturer’s instructions. The RNA-seq transcriptome library was prepared following Illumina^®^ Stranded mRNA Prep, Ligation (San Diego, CA) using 1μg of total RNA. the sequencing library was performed on NovaSeq X Plus platform (PE150) using NovaSeq Reagent Kit.

The raw paired end reads were trimmed and quality controlled by fastp with default parameters. Then clean reads were separately aligned to reference genome with orientation mode using HISAT2 software. The mapped reads of each sample were assembled by StringTie in a reference-based approach.

To identify DEGs (differential expression genes) between two different samples, the expression level of each transcript was calculated according to the transcripts per million reads (TPM) method. RSEM[4] was used to quantify gene abundances. Essentially, differential expression analysis was performed using the DESeq2 or DEGseq. DEGs with |log2FC|≧1 and FDR< 0.05(DESeq2) or FDR < 0.001(DEGseq) were considered to be significantly different expressed genes. In addition, functional-enrichment analysis including GO and KEGG were performed to identify which DEGs were significantly enriched in GO terms and metabolic pathways at Bonferroni-corrected P-value < 0.05 compared with the whole-transcriptome background. GO functional enrichment and KEGG pathway analysis were carried out by Goatools and Python scipy software, respectively.

### 2.8 Multiplex cytokine profiling and enzyme-linked immunosorbent assays

Cytokines and chemokines secreted by NO-infected and AO-infected were measured and analyzed using the Bio-Plex Pro human Chemokine Panel (Bio-Rad) according to the manufacturer’s instructions. The kits used in this study included the human cytokine panel with Eotaxin, MIP-3beta, MCP-1, MIP-3alpha, PD-L1/B7-H1, MIP-1alpha, MIP-1beta, CD40 Ligand, GRO alpha, IP-10, GRO beta, IL-8, EGF, IFN-gamma, FIt-3 Ligand, G-CSF, GM-CSF, Granzyme B, IFN-alpha2, IFN-beta, TNF-beta, IL-10, IL-12p70, IL-13, IL-15, IL-17A, IL-17E, IL-9, IL-1beta, IL-1ra, IL-2, IL-3, IL-33,IL-4, IL-5, IL-6, IL-7, PDGF-AA, PDGF-AB/BB, RANTES, TGF-alpha, TNF-alpha, TRAIL, VEGF, FGF basic, IL-1alpha. Data were obtained with Luminex X-200 and analyzed with MILLIPLEX Analyst v5.1.0.0 standard build.

IFNa, IFNβ, IFN-γ levels in culture supernatants were determined with ELISA kits from R&D Systems(shanghai, China, Catalog No.VAL169,VAL137) or RayBiotech (Catalog No. ELH-IFNg) according to the manufacturer’s instructions.

### 2.9 High-speed microscopy analysis of the ciliary beating frequency

The NO and AO were prepared at room temperature (25°C) for video microscopy with a 40× objective (Sprinter-HD Optronics) to eliminate the influence of environmental factors such as temperature. Three organoid model field images were available for each sample, and movies were recorded at 200 fps to record the ciliary beating frequency (CBF), which was analyzed blindly by two researchers. Kymographs of ciliary beating were depicted with a macro embedded in ImageJ (**Supplementary Figure 3**).

### 2.10 Statistical analysis

Each experiment was replicated at least three times. Statistical analyses of the demographic data were performed via Prism (version 8.3.0, GraphPad Software, San Diego, California). To compare the differences between the two groups, t tests were used for normally distributed data, whereas Wilcoxon matched-pairs signed rank tests were used for nonnormally distributed data. For comparisons between multiple groups, one-way and two-way analysis of variance (ANOVA) followed by Dunnett’s multiple comparisons test was used. The significant differences between the groups were denoted by * p < 0.05.

## 3 Results

### 3.1 Insufficient expression of endogenous type I and II interferon proteins in nasal and airway epithelial cells of children following RSV infection

To investigate whether RSV triggers an innate immune response in children’s nasal and airway epithelium, we constructed the organoids and compared differences in the expression levels of various endogenous interferons in pediatric nasal and airway organoids before and after RSV infection. The highly differentiated NO and AO form hollow spheres (**Supplementary Figure 1A**), with no significant difference in diameter between the two types (**Supplementary Figure 1C**). Immunofluorescence staining confirmed the expression of all four cell types in the epithelium of both organoids under steady-state conditions (**Supplementary Figure 1D**). However, high-speed camera recordings of ciliary beating revealed that airway epithelial cilia beat at a higher frequency than nasal epithelial cilia (**Supplementary Figure 1B**), indicating structural differences between the two epithelial types.

**Figure 1:**
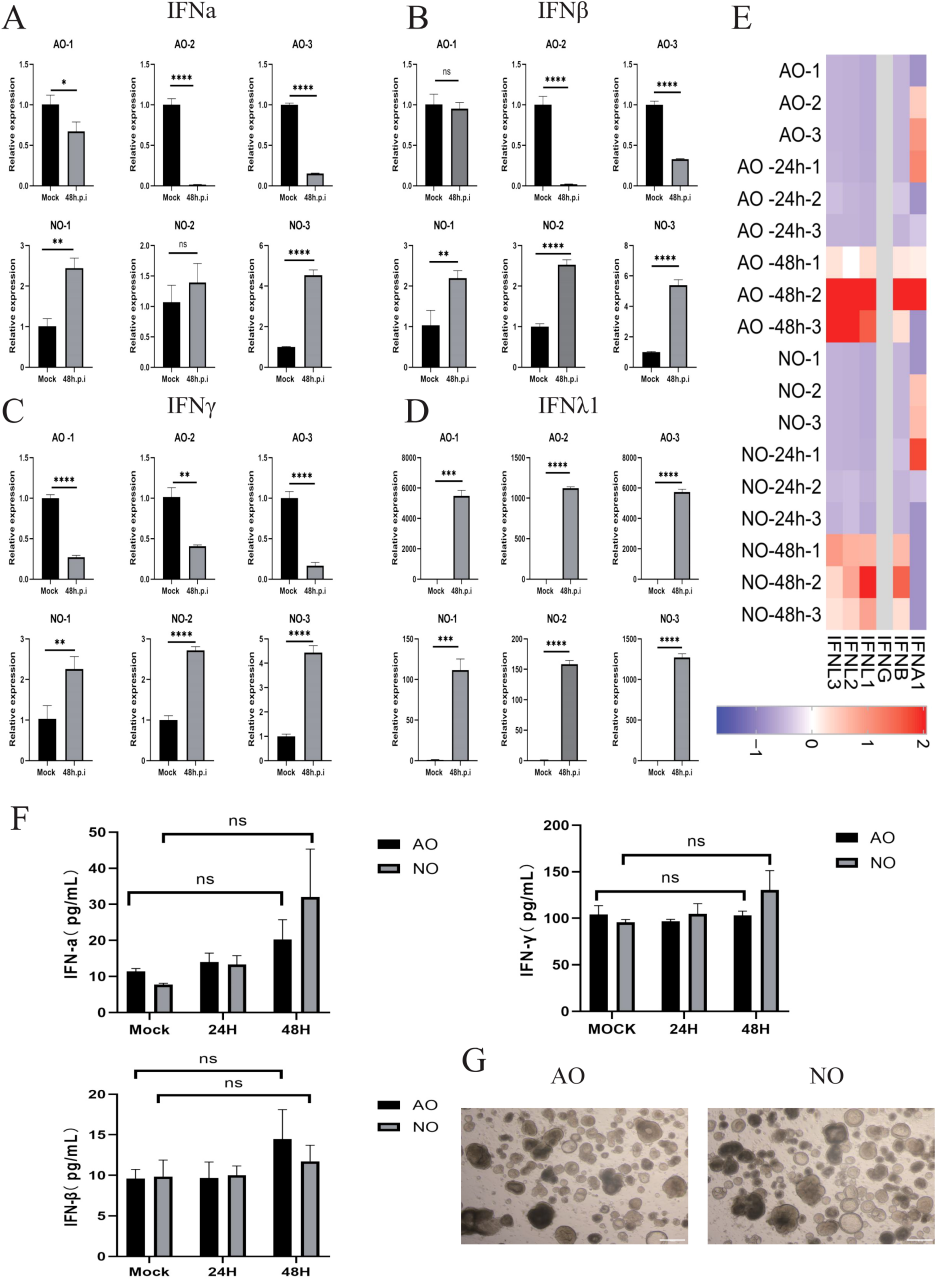
Following RSV infection from different donors, children exhibited upregulated type I, II, and III interferon expression in the nasal epithelium, whereas the airway epithelium showed downregulation of type I and II interferons. **(A)** Pre- and post-RSV infection IFNα gene expression levels in children’s airway and nasal epithelium. **(B)** Pre- and post-RSV infection IFNβ gene expression levels in children’s airway and nasal epithelium. **(C)** IFNγ gene expression levels in pediatric airway and nasal epithelial before and after RSV infection. **(D)** IFNλ1 gene expression levels in pediatric airway and nasal epithelial before and after RSV infection. **(E)** Heatmap of gene transcripts in pediatric airway and nasal epithelial cells before and after RSV infection. Red indicates upregulation, blue indicates downregulation, and gray indicates near-zero expression. **(F)** Protein expression levels of type I and II IFN in pediatric airway and nasal epithelial before and after RSV infection. **(G)** Microscopic image of RSV-infected pediatric airway and nasal epithelial after RSV infection. Scale bar: 500 μm. Human airway epithelial organoids-1 (AO-1), Human airway epithelial organoids-2 (AO-2), Human airway epithelial organoids-3 (AO-3), Human nasal epithelial organoids-1 (NO-1), Human nasal epithelial organoids-2 (NO-2), Human nasal epithelial organoids-3 (NO-3). n = 3 biological replicates, each with 3 technical replicates. * p < 0.05, ** p < 0.01, ***p < 0.001, ****p < 0.0001.

Notably, RSV infection resulted in an increased expression of endogenous type I and II interferon mRNA expression in pediatric nasal epithelial organelles, while decreasing endogenous type I and II interferon mRNA expression in airway epithelial organelles (**Figures 1A–C**). Endogenous type III interferon mRNA expression levels significantly increased in both pediatric nasal and airway organoids subsequent to RSV infection (**Figure 1D**). Transcriptome sequencing further corroborated these findings, indicating elevated expression of IFNβ and IFNλ in nasal and airway epithelial following RSV infection (**Figure 1E**). To further characterize the expression levels of type I and II interferon proteins, we conducted ELISA assays. Results demonstrated that endogenous type I and II interferon protein expression in pediatric nasal and airway organoids exhibited certain limitations 48 hours after RSV infection (**Figure 1F**). Under bright-field microscopy, we observed significant morphological changes in both nasal and airway organoids 48 hours post-RSV infection. Organoids transformed from hollow to solid structures, exhibiting deformation and distortion (**Figure 1G**).

### 3.2 Exogenous treatment with all four interferons reduces RSV viral load in nasal and airway epithelial cells

**Figure 2:**
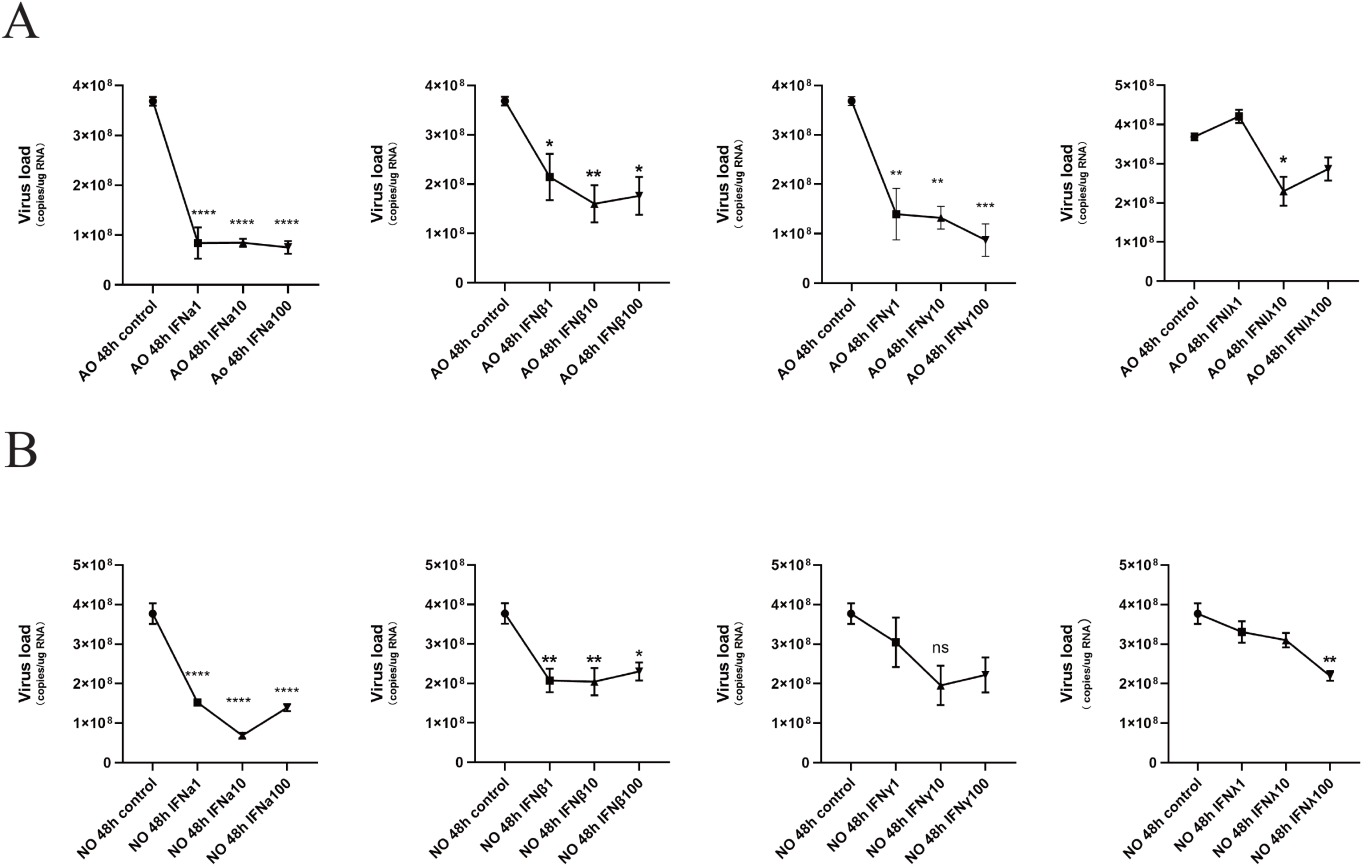
All four types of interferons reduce RSV viral load in nasal and airway epithelial cells. **(A)** The ddPCR measured RSV viral load in airway epithelial cells treated with three concentration gradients (1 ng/ml, 10 ng/ml, 100 ng/ml) of each of the four exogenous interferons (IFNa, IFNβ, IFNγ, IFNλ1) and the control group. **(B)** The ddPCR measured RSV viral load in nasal epithelium following treatment with three concentration gradients (1 ng/ml, 10 ng/ml, 100 ng/ml) of each of four exogenous interferons (IFNa, IFNβ, IFNγ, IFNλ1) and the control group. * p < 0.05, ** p < 0.01, ***p < 0.001, ****p < 0.0001.

To assess whether exogenous interferon provides protection against RSV in pediatric nasal and airway epithelial models, and how this protection is influenced by the specific interferon type, dosage, and site of application. We divided pediatric nasal and airway epithelial organoids into control and intervention groups and infected with RSV for 48 hours. The intervention group received three concentration gradients (1 ng/ml, 10 ng/ml, 100 ng/ml) of four exogenous interferons (IFNa, IFNβ, IFNγ, IFNλ1) added to complete culture medium, while the control group received equivalent volumes of DPBS. Digital PCR results indicated that exogenous treatment with the four interferons (IFNa, IFNβ, IFNγ, IFNλ1) conferred protective effects on both pediatric nasal and airway epithelial cells. Digital PCR indicated that IFNα (100 ng/ml), IFNβ (10 ng/ml), IFNγ (100 ng/ml), and IFNλ1 (10 ng/ml) significantly reduced viral load in pediatric airway organoids (**Figure 2A**). IFNα (10 ng/ml), IFNβ (10 ng/ml), IFNγ (10 ng/ml) and IFNλ1 (100 ng/ml) significantly reduced viral load in pediatric nasal organoids (**Figure 2B**).

### 3.3 Type I IFN specifically suppresses proinflammatory cytokine expression while preserving IFN-dependent antiviral responses

**Figure 3:**
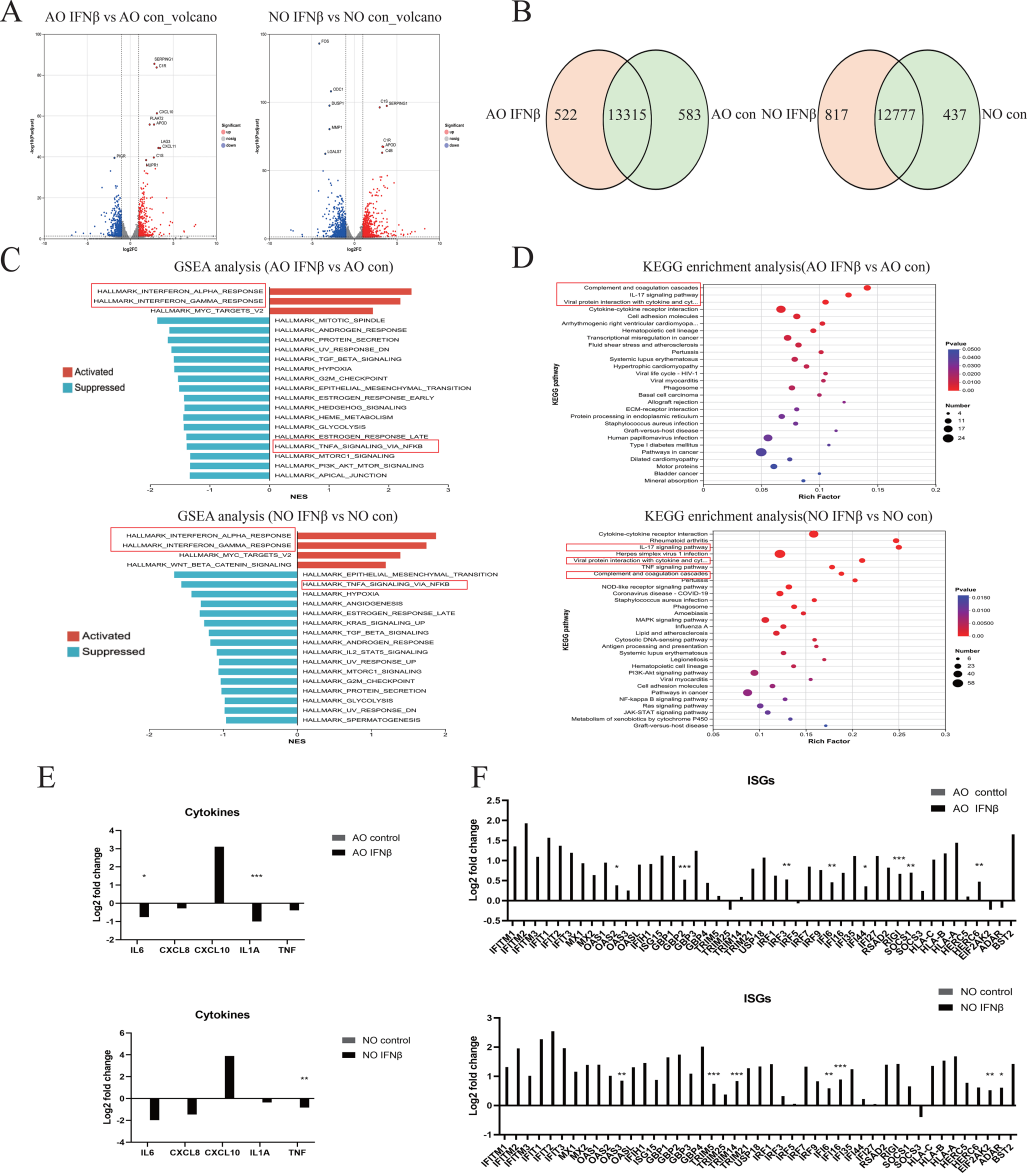
Host Type I Interferon Transcriptional Response in Airway and Nasal Organoids. RNA sequencing analysis of RSV-infected airway organoids (AO) and nasal organoids (NO) treated with type I interferon or DPBS. **(A)** Volcano plot showing genes upregulated and downregulated in type I interferon-treated airway and nasal organoids compared to controls. **(B)** The Venn diagram shows the number of genes shared and unique to airway and nasal organoids treated with type I interferon and the control group. **(C)** GSEA plot showing the top 20 up- and downregulated pathways in type I interferon-treated airway and nasal organoids compared to controls. **(D)** KEGG plot showing the top 30 pathways in type I interferon-treated airway and nasal organoids compared to controls. **(E)** RSV-relevant pro-inflammatory cytokines. **(F)** IFN response genes normalized to the infected control. Data are expressed as means of Log2-foldchanges. *p < 0.05, **p < 0.01, ***p < 0.001.

Next, to delineate the mechanism of type I interferon in pediatric nasal and airway epithelium, we performed RNA sequencing with KEGG and GSEA analyses. Volcano plots showed that IFNβ upregulates complement classical pathway components C1R and C1S in both epithelial types(**Figure 3A**). The Venn diagram revealed 13,315 shared genes in airway epithelium (583 unique to control, 522 unique to IFNβ), and 12,777 shared genes in nasal epithelium (437 unique to control, 817 unique to IFNβ) (**Figure 3B**). KEGG analysis indicated enrichment in the complement and coagulation cascades, IL-17 signaling pathway, and viral proteins interacting with cytokines-cytokine receptors (**Figure 3D**). GSEA analysis showed that IFNβ upregulated IFNα and IFNγ pathways but downregulated the TNFα–NFκB pathway (**Figure 3C**). Consistent with this, IFNβ treatment reduced mRNA levels of inflammatory cytokines (including IL6, CXCL8, IL-1a, TNF) except CXCL10 (**Figure 3E**), while elevating interferon-stimulated genes (ISGs) (**Figure 3F**). These results confirm that type I interferon suppresses pro-inflammatory cytokine expression while maintaining antiviral responses.

### 3.4 Type II IFN broadly increased mRNA expression levels of ISGs in pediatric nasal epithelium, but partially increased ISG mRNA expression in pediatric airway epithelium

**Figure 4:**
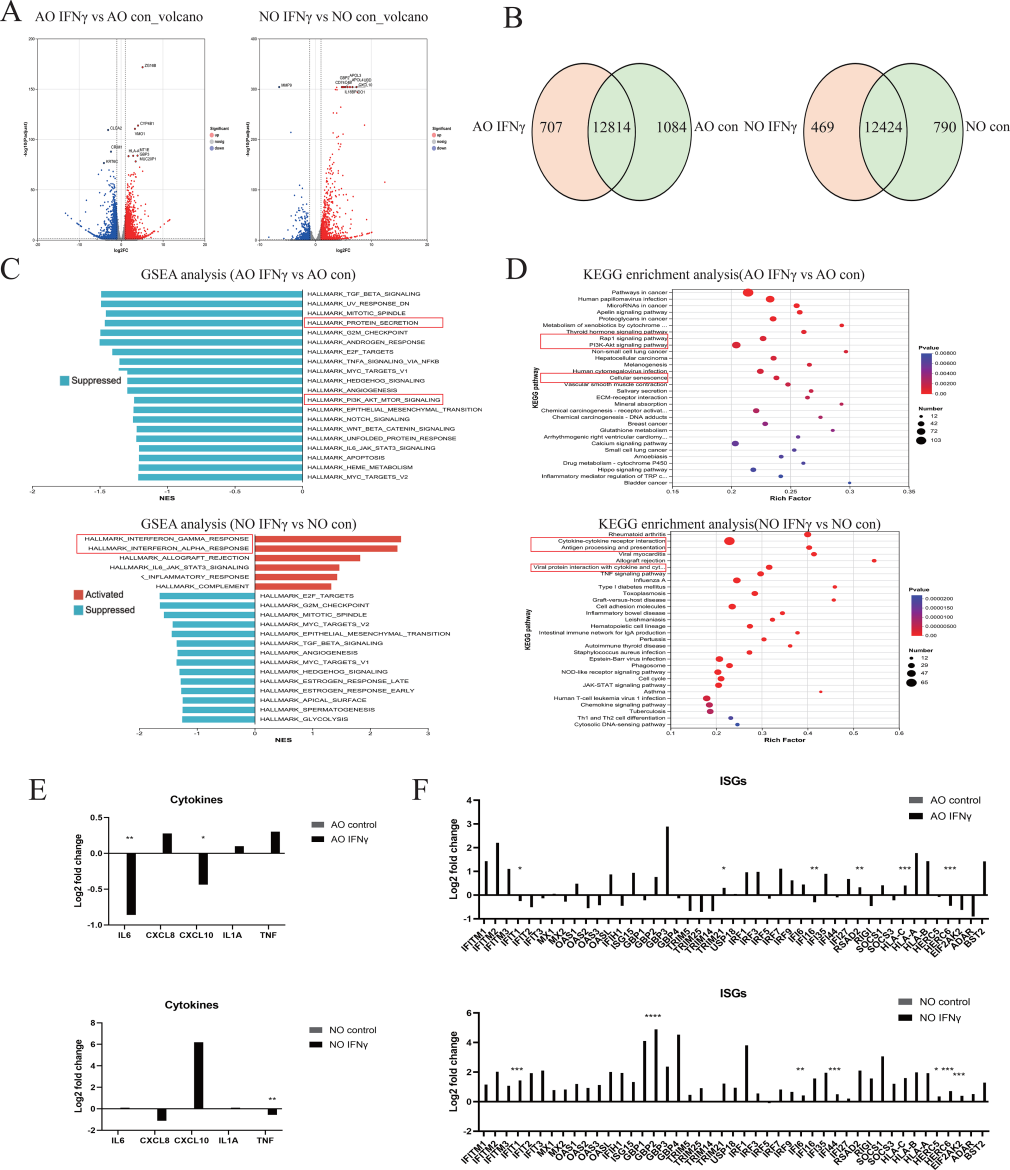
Host Type II Interferon Transcriptional Response in Airway and Nasal Organoids. RNA sequencing analysis of RSV-infected airway organoids (AO) and nasal organoids (NO) treated with type II interferon or DPBS. **(A)** Volcano plot showing genes upregulated and downregulated in type II interferon-treated airway and nasal organoids compared to controls. **(B)** The Venn diagram shows the number of genes shared and unique to airway and nasal organoids treated with type II interferon and the control group. **(C)** GSEA plot showing the top 20 upregulated and downregulated pathways in type II interferon-treated airway and nasal organoids compared to controls. **(D)** KEGG diagram showing the top 30 pathways in type II interferon-treated airway and nasal organoids compared to controls. **(E)** RSV-relevant pro-inflammatory cytokines. **(F)** IFN response genes normalized to the infected control. Data are expressed as means of Log2-fold changes. *p < 0.05, **p < 0.01, ***p < 0.001, ****p < 0.0001.

Similarly, to understand the mechanism of type II interferon in pediatric nasal and airway epithelium, we performed RNA sequencing with KEGG and GSEA analyses. Volcano plots showed that IFNγ upregulates human major histocompatibility complex components (HLA-A, CD74) and interferon-stimulated genes (GBP3, GBP2) in airway and nasal epithelium (**Figure 4A**). The Venn diagram revealed 12,814 shared genes in airway epithelium (1,084 unique to control, 707 unique to IFNγ), and 12,424 shared genes in nasal epithelium (469 unique to control, 790 unique to IFNγ) (**Figure 4B**). KEGG analysis indicated that in airway epithelium, IFNγ primarily enriched the Rap1 signaling pathway, PI3K-Akt signaling pathway and cellular senescence, while in nasal epithelium it mainly enriched cytokine-cytokine receptor interactions, antigen processing and presentation and viral proteins interacting with cytokines-cytokine receptors(**Figure 4D**). GSEA analysis showed that IFNγ upregulated IFNα and IFNγ signaling pathways in nasal epithelium, but downregulated the protein secretion pathway and PI3K-Akt-mtor pathway in airway epithelium (**Figure 4C**). Accordingly, IFNγ treatment upregulated CXCL10 in nasal organoids, while airway organoids exhibited increased mRNA expression of CXCL8, IL-1a, and TNF (**Figure 4E**). Interestingly, IFNγ broadly increased ISG mRNA expression in nasal epithelium, but only partially increased ISGs in airway epithelium (**Figure 4F**).

### 3.5 Type III IFN targets the suppression of proinflammatory cytokine expression while preserving IFN-dependent antiviral responses

**Figure 5:**
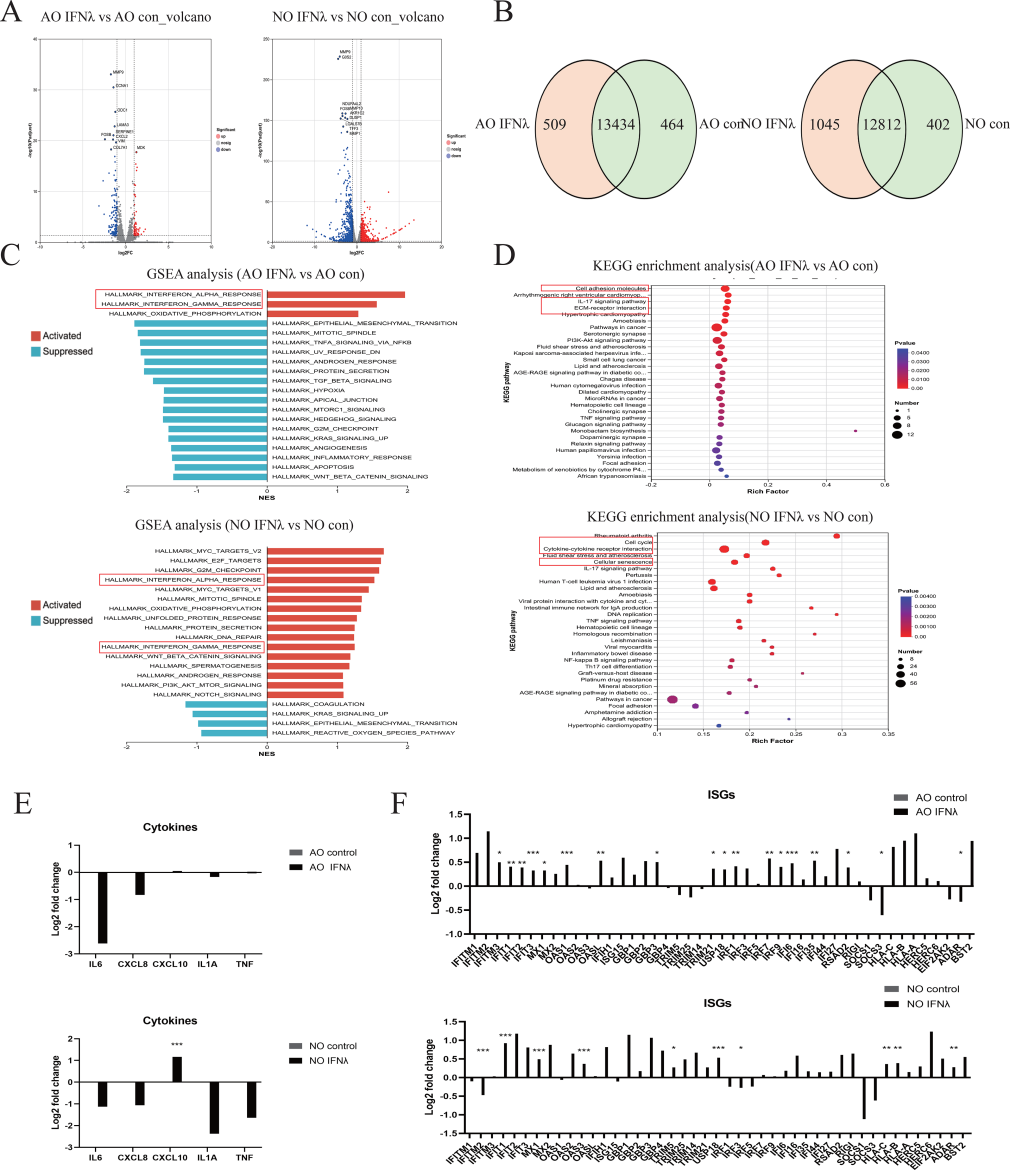
Host Type III Interferon Transcription Response in Airway and Nasal Organoids. RNA sequencing analysis of RSV-infected airway organoids (AO) and nasal organoids (NO) treated with type III interferon or DPBS. **(A)** Volcano plot showing genes upregulated and downregulated in type III interferon-treated airway and nasal organoids compared to controls. **(B)** The Venn diagram shows the number of genes shared and unique to airway and nasal organoids treated with type III interferon and the control group. **(C)** GSEA plot showing the top 20 upregulated and downregulated pathways in type III interferon-treated airway and nasal organoids compared to controls. **(D)** KEGG diagram showing the top 30 pathways in type III interferon-treated airway and nasal organoids compared to controls. **(E)** RSV-relevant pro-inflammatory cytokines. **(F)** IFN response genes normalized to the infected control. Data are expressed as means of Log2-fold changes. *p < 0.05, **p < 0.01, ***p < 0.001.

Finally, to elucidate the mechanism of type III interferon in pediatric nasal and airway epithelium, we performed RNA sequencing with KEGG and GSEA analyses. Volcano plots showed that IFNλ1 downregulates matrix metalloproteinase (MMP9) and cellular stress molecule (FOSB) in both epithelial types (**Figure 5A**). The Venn diagram revealed 13,434 shared genes in airway epithelium (464 unique to control, 509 unique to IFNλ1), and 12,812 shared genes in nasal epithelium (402 unique to control, 1,045 unique to IFNλ1) (**Figure 5B**). KEGG analysis indicated that in airway epithelium, IFNλ1 primarily enriched cell adhesion molecules, IL-17 signaling pathway and ECM-receptor interaction, while in nasal epithelium it mainly enriched cell cycle, cytokine-cytokine receptor interaction and cellular senescence (**Figure 5D**). GSEA analysis showed that IFNλ1 upregulated IFNα and IFNγ signaling pathways in both epithelial types (**Figure 5C**). Accordingly, IFNλ1 treatment decreased inflammatory cytokines (including IL6, CXCL8, IL-1a, TNF) except CXCL10 (**Figure 5E**), while elevating interferon-stimulated genes (ISGs) (**Figure 5F**). These results confirm that type III interferon also suppresses pro-inflammatory cytokine expression while maintaining antiviral responses.

### 3.6 IFN-λ1 shows the greatest anti-inflammatory effect in nasal epithelium, while IFN-β doesn’t exacerbate inflammation in airway epithelium

To characterize host-specific changes in cytokine secretion during RSV infection, we performed a 46-cytokine Luminex assay on untreated and interferon-treated groups 48 hours post-RSV infection. The innate inflammatory response to RSV infection plays a critical role in determining disease severity, as excessive inflammation characterized by increased production of proinflammatory cytokines and chemokines is associated with more severe RSV disease, prolonged illness, and increased risk of secondary infections. To further explore inflammatory factor alterations, inflammatory factors were functionally categorized into 11 groups: chemokines, proinflammatory factors, regulatory factors, anti-inflammatory factors, hematopoietic stem cell response, allergic response, cell proliferation, antiviral response, apoptosis, Th1 response and angiogenesis.

**Figure 6:**
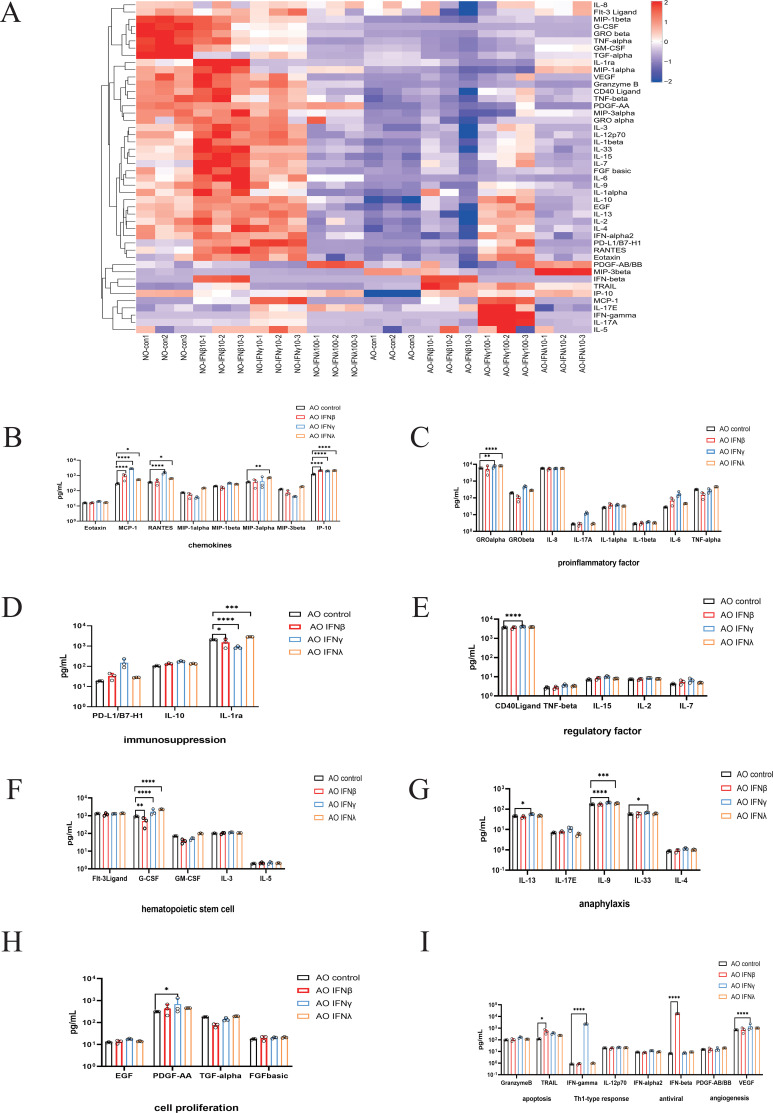
Cytokine Responses in Control and Interferon-Treated Groups During RSV Infection in Pediatric Airway Epithelial Organoids. **(A)** Heatmap showing overall cytokine expression between control and interferon-treated groups during RSV infection in pediatric nasal and airway epithelial organoids. Inflammatory factors were functionally categorized into 11 groups: chemotactic factors, pro-inflammatory factors, regulatory factors, anti-inflammatory factors, hematopoietic stem cell response, allergic response, cell proliferation, antiviral response, apoptosis, Th1 response and angiogenesis. Bar charts show differences in cytokine expression between the control and interferon-treated groups during RSV infection in pediatric airway epithelial organoids. **(B)** Chemotactic factors. **(C)** pro-inflammatory factors. **(D)** anti-inflammatory factors. **(E)** regulatory factors. **(F)** hematopoietic stem cell response factors. **(G)** allergic response factors. **(H)** cell proliferation factors. **(I)** antiviral response, apoptosis, Th1 response and angiogenesis. *p < 0.05, **p < 0.01, ***p < 0.001, ****p < 0.0001.

Statistical analysis revealed distinct cytokine responses to interferon treatment in pediatric nasal and airway epithelium. For nasal epithelium (**Supplementary Figure 2**), IFN-λ1 treatment resulted in the most widespread suppression, significantly reducing levels of chemokines (MIP-1β, MIP-3α, MCP-1, RANTES), proinflammatory factors (GRO-β, TNF-α), hematopoietic factors (G-CSF, GM-CSF), and the cell proliferation factor TGF-α, along with angiogenesis factor VEGF. In contrast, IFN-β and IFN-γ increased the chemokines MCP-1 and RANTES, but suppressed MIP-1β, G-CSF, GM-CSF, and TGF-α. The anti-inflammatory factor IL-1α was downregulated by IFN-γ and IFN-λ but upregulated by IFN-β. For airway epithelium, IFN-γ triggered the most widespread response, significantly upregulating chemokines (MCP-1, RANTES), proinflammatory factors (GRO-α, GRO-β, IL-17A, IL-6), allergic response factors (IL-13, IL-17E, IL-9, IL-33, IL-4), and the hematopoietic factor G-CSF, while reducing the anti-inflammatory factor IL-1α. IFN-λ1 triggered a more targeted response, elevating a set of factors that included IP-10, MIP-3α, MIP-3β, GRO-α, and G-CSF. In contrast, IFN-β had a slight effect, increasing only IP-10 and the apoptosis factor TRAIL, while reducing G-CSF (**Figure 6B–I**).

Our data also demonstrated that the nasal epithelium mounted a more robust cytokine response than the airway epithelium following RSV infection (**Figure 6A**). In short, IFN-λ1 reduced the overall cytokine response in the nasal epithelium, whereas airway epithelium treated with IFN-β triggered only a minimal cytokine response.

## 4 Discussion

Our study reveals that pediatric RSV infection triggers an inadequate endogenous type I and II interferon response in both the upper and lower respiratory tracts. Exogenous supplementation with interferons consistently reduced viral load. IFN-λ1 is preferable for the nasal epithelium, while IFN-β is optimal for the airway epithelium. Mechanistically, both type I and III IFNs selectively suppressed the expression of pro-inflammatory cytokines while preserving the IFN-dependent antiviral state. In contrast, type II IFN strongly upregulated interferon-stimulated genes (ISGs) in the upper respiratory tract but had limited effects in the lower tract. The upper respiratory tract exhibited a more robust immune response at 48 hours post-infection, and the anti-inflammatory effects of interferons were tissue-specific: IFN-λ1 could reduce the inflammatory response in the nasal epithelium, whereas IFN-β did not exacerbate inflammatory responses in the airway epithelium.

In viral infections, interferons are key players in the first line of defense. They help prevent viruses from entering cells, block viral replication, and attract immune cells (37, 38). Type I (IFN-α/β) and type III (IFN-λ) interferons are especially known for their antiviral roles (39, 40). Although IFN-γ is a hallmark of T cell-mediated adaptive immunity, it also plays a less-defined role in antiviral defense. Studies have demonstrated that a strong IFN-α response is associated with protective effects and milder disease progression in infants with RSV infection (41). Unfortunately, it is well established that RSV suppresses the innate immune response in infants, particularly the production of type I interferons, which may contribute to severe disease progression (42). Furthermore, RSV-induced IFN-α production, which is primarily mediated by plasmacytoid dendritic cells (pDCs), increases with age (43). IFN-β is a key component of the immune response to RSV infection, regulating immunity and suppressing viral replication (44). Notably, Van et al. demonstrated that RSV establishes mechanisms to attenuate the host IFN-β response (45). IFN-γ stimulates antigen presentation by inducing the expression of major histocompatibility complex (MHC) molecules and enhances the cytotoxic activity of virus-specific natural killer (NK) cells and T cells. The early IFN-γ response plays a crucial role in influencing the course of viral infection. Therefore, reduced IFN-γ production during RSV infection may be a determinant of disease severity. Isolated whole-blood cultures stimulated with RSV antigens exhibited a lack of robust IFN-γ secretion and IFN-γ mRNA expression (46). Eichinger et al. provided evidence in animal models that treatment with IFN-γ can prevent RSV infection (47). IFN-λ is one of the primary interferons produced in the airways during respiratory viral infection. Its receptor is mostly found on epithelial cells, making it particularly relevant at mucosal surfaces. RSV infection induces high levels of IFN-λ 1–3 expression in the lungs (48). IFN-λ is the primary IFN secreted by RSV-infected nasal epithelial cells (NECs), a process dependent on RIG-I recognition (49, 50).

Type I and III interferons exhibit strong functional similarities, demonstrating potent antiviral activity, engaging overlapping signaling pathways, and consistently reducing pro-inflammatory mediators (IL-6, IL-1α, TNF, CXCL8) —while enhancing interferon-stimulated genes (ISGs) expression in both nasal and airway epithelium. In contrast, IFN-γ showed efficacy in nasal epithelium but was less effective in airway epithelium, possibly due to its role in the adaptive immune system, which is underdeveloped in young children, unlike the innate immune origin of type I/III IFNs. The adaptive immune system in children is not fully developed, with deficiencies in T-cell number, activation, proliferation, and migration. Airway immune responses largely depend on systemic adaptive immunity, requiring the recruitment of activated effector T cells (e.g., CD8^+^ T cells) from the circulation and lymphoid tissues to the local site; thus, exogenous IFN-γ exhibits limited efficacy. This is also reflected in the Th1/Th2 imbalance observed in children with RSV lower respiratory tract infection (51). On the other hand, studies such as that by Uddbäck I et al. have shown that IFN-γ upregulates MHC-I and MHC-II expression in murine nasal epithelium, indicating its clear antiviral role in nasal tissue (52). In contrast, type I/III interferons (IFN-α/β, IFN-λ) act directly on all nucleated cells, including epithelial cells, initiating an innate antiviral program independent of T cells, thereby still providing effective immune protection in the pediatric airway. This supports the tissue-specific utility of IFN subtypes.

Our study identifies distinct interferon response patterns to RSV infection in pediatric respiratory tracts: type III (IFN-λ1) responses were strong, while type I and II responses were subdued. Notably, high mRNA levels did not correlate with protein secretion, suggesting post-transcriptional inhibition—including in mRNA stability, translation efficiency, or protein degradation (53) —and highlights the need for exogenous interferon supplementation. Additionally, despite elevated mRNA levels, the immune system may possess regulatory mechanisms that limit excessive IFN protein production and secretion to prevent harmful overactivation of the inflammatory response. Further analysis of post-transcriptional mechanisms and secretion studies may help elucidate these issues. Transcriptome analysis revealed IFN-specific pathway alterations: Type I IFN enriched complement and coagulation cascades, IL-17 signaling pathway, and viral proteins interacting with cytokines and cytokine receptors in both nasal and airway epithelium. Type II IFN enriched Rap1 signaling pathway, PI3K-Akt signaling pathway, and cellular senescence in airways, but enriched cytokine-cytokine receptor interactions, antigen processing and presentation, and viral proteins interacting with cytokines-cytokine receptors in nasal epithelium. Type III IFN enriched cell adhesion molecules, IL-17 signaling pathway, and ECM-receptor interaction in airways, while enriched cell cycle, cytokine-cytokine receptor interactions, and cellular senescence in nasal epithelium. We suggest that IFN-γ enriches the “antigen presentation” pathway in nasal epithelium but the “cellular senescence” pathway in airway epithelium. This likely results from their different anatomical locations and immune environments. In nasal epithelium—a first-line defense site rich in immune cells—IFN-γ mainly activates immune cells and promotes antigen presentation. In lower airway epithelium, under strong stimuli such as infection, IFN-γ directly acts on epithelial cells, leading to senescence-like changes, hyperplasia, and fibrosis. This view is supported by earlier findings: antigen presentation increases in nasal epithelium after influenza infection (54), and airway epithelium in COPD shows a type II IFN–driven senescent phenotype (55). Type I IFN enriched complement and coagulation cascades in both nasal and airway epithelium. Volcano plots revealed that IFN-β upregulates key classical pathway components C1R and C1S in both epithelial types, indicating that IFN-β primarily activates the classical complement pathway. This pathway initiates when C1q binds directly to a pathogen’s surface or to antibody-bound immune complexes, leading to the activation of serine proteases C1r and C1s, assembly of the C1 complex, and subsequent cleavage of C4 and C2 to form the C3-convertase (C4b2a) (56). As an essential part of the immune system, complement enhances viral clearance by antibodies, promotes phagocytosis of viral particles, lyses infected cells, and recruits immune cells. A similar protective role of complement has been demonstrated in infections such as HBV (57) and IAV (58).

Following viral entry, both extracellular and intracellular pattern recognition receptors (PRRs) detect the invading virus and trigger the innate immune response. PRRs are primarily categorized into three families: Toll-like receptors (TLRs), NOD-like receptors (NLRs), and RIG-I-like receptors (RLRs). Among them, TLR2, TLR3, TLR4, TLR6, TLR7, RIG‐I, and NOD2 can recognize pathogen-associated molecular patterns (PAMPs) from RSV.TLRs are present on a diverse range of cells, including macrophages, dendritic cells, epithelial cells, neutrophils, and eosinophils. In humans, 11 distinct TLRs have been identified. Surface TLRs such as TLR1, TLR2, TLR4, TLR5, and TLR6 engage with RSV surface molecules (eg LPS or RSV fusion[[F] proteins).In contrast, TLR3, TLR7, TLR8, and TLR9 reside within intracellular vesicles, where they sense viral nucleic acids.TLR4, the most studied and significant PRR, recruits myeloid differentiation primary response protein 88 (MyD88) and TIR-domain-containing adapter-inducing interferon-β (TRIF), which allows interferon regulatory factor (IRF) or nuclear factor kappa‐B (NF‐kB) into the nucleus, driving the expression and secretion of proinflammatory cytokines, interferon, and TNF, thereby eliciting an inflammatory state (59). Genetic variations in TLR4 are associated with heightened susceptibility to RSV infection, and studies in TLR4-deficient animal models show delayed viral clearance (60). The RLR family, comprising cytosolic sensors like RIG-I and MDA5, detects viral RNA. Upon binding to pathogen-derived ssRNA or dsRNA, they interact with the mitochondrial antiviral-signaling protein (MAVS) (61), activating downstream pathways that induce the synthesis of IFN and proinflammatory cytokines. Secreted interferons then act in an autocrine or paracrine manner by binding to their cognate receptors and activating the JAK-STAT signaling pathway. Phosphorylated STAT proteins translocate to the nucleus and induce the transcription of hundreds of interferon-stimulated genes (ISGs). The proteins encoded by these ISGs exert broad antiviral effects by targeting multiple stages of the viral replication cycle—such as degrading viral mRNA, inhibiting viral protein translation, and suppressing the initiation of viral protein synthesis (62). Clinical evidence indicates a significant positive correlation between RSV viral load and RIG-I mRNA expression in infants with RSV bronchiolitis (63). Furthermore, RSV challenge in healthy volunteers upregulates RIG-I expression, underscoring its pivotal role in the anti-RSV immune response. NLRs function as cytoplasmic sensors for ssRNA and regulate diverse processes like inflammasome assembly, transcription, and autophagy. During RSV infection, the NLR member NOD2 recognizes viral ssRNA and promotes the production of IRF3 and IFN via the NF-κB pathway. NOD2-deficient mice exhibit attenuated IRF3 activation and IFN-I production, resulting in increased severity of RSV-induced lung pathology (64).

From a comparative perspective, we further observed that nasal epithelial cells generally exhibit stronger immune reactions than airway cells from the same patient. Other researchers have reported similar patterns, supporting the idea that nasal epithelium often mounts a stronger antiviral response than bronchial epithelium. Mihylova et al. reported that primary nasal epithelial cells mounted stronger antiviral responses than bronchial epithelial cells following rhinovirus B infection or RIG-1 stimulation (65). However, the individual analysis of virus-infected cells and Poly(I:C) stimulation consistently indicated higher levels of immune activation in airway organoids compared to nasal organoids (66). Since a paired experimental design was employed, the observed effects are not confounded by inter-individual variability, thereby strengthening the validity of our findings.

Severe RSV infection promotes a pro-inflammatory environment through the production of cytokines and chemokines such as IL-6, IL-8, IL-11, and monocyte chemotactic protein (MCP)-1a, which increase bronchial hyperresponsiveness (67). Based on the tissue-specific anti-inflammatory profiles of interferons, we recommend IFN-λ1 for the upper airways, as it potently suppressed multiple inflammatory mediators, including GRO-β, TNF-α, MIP-1β, MIP-3α, MCP-1, RANTES, G-CSF, GM-CSF, TGF-α, and VEGF. In contrast, IFN-β is proposed for the lower airways due to its mild immunomodulatory activity—only elevating IP-10 and TRAIL-thus avoiding the broad pro-inflammatory response seen with IFN-γ, which upregulates MCP-1, RANTES, IL-6, IL-13, IL-4, and other allergy-related factors. Nebulized interferon delivery offers several advantages: (1) Rapid onset through direct mucosal application; (2) Targeted action via local receptor engagement; (3) Minimized systemic exposure with additional benefits of airway humidification and sputum thinning; and (4) Improved pediatric compliance compared to intramuscular injections. Chen et al. proved that nebulized IFN-α is effective in treating bronchiolitis (28). Our findings still have obvious limitations. First, while our organoid model recapitulates certain 3D structural and multicellular features, it remains primarily an epithelial system and cannot fully mimic the complex *in vivo* microenvironment—which includes vasculature, stroma, and immune components. Second, the drug concentration used *in vitro* may differ from the physiologically relevant concentration *in vivo* due to factors such as tissue penetration and metabolism. Future investigations using animal models and clinical trials are therefore essential to substantiate these preliminary findings.

## 5 Conclusion

Based on our study, pediatric nasal and airway organoids demonstrate distinct interferon (IFN) responses to RSV infection. Both type I and type III IFNs effectively reduce viral load and suppress key pro-inflammatory cytokines while preserving antiviral interferon-stimulated genes (ISGs) expression. The nasal epithelium exhibits a stronger immune response than the airway epithelium. These findings highlight the importance of selecting the appropriate interferon type and delivery site, suggesting thatIFN-λ1 could be optimal for the upper airways and IFN-β for the lower airways, providing a strategic basis for optimizing interferon therapy in children.

## Data Availability

The datasets presented in this study can be found in online repositories. The names of the repository/repositories and accession number(s) can be found in the article/**Supplementary Material**.
