# Supplementary figures and images for: Differential antiviral effects and immune responses in nasal and airway organoid during RSV infection: implications for interferon therapy
Source: Front Immunol. 2026 Feb 18;17:1754206. doi: 10.3389/fimmu.2026.1754206 (PMC12957070; doi:10.3389/fimmu.2026.1754206)

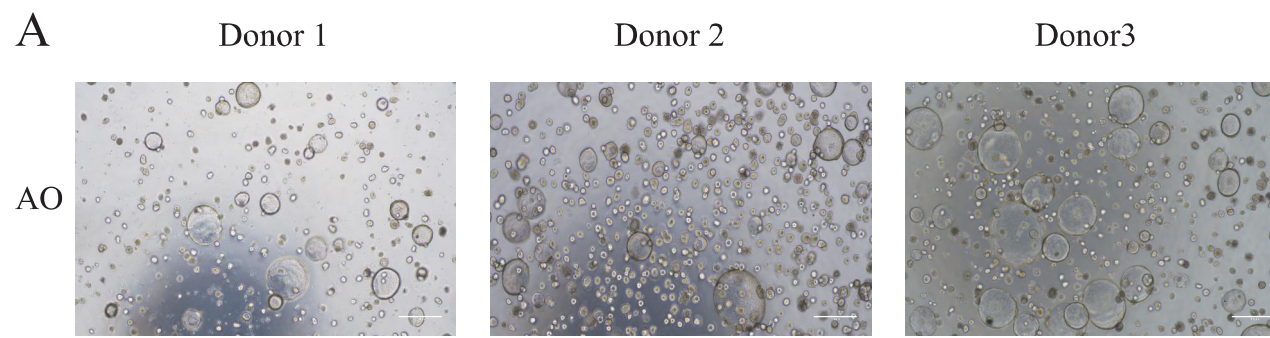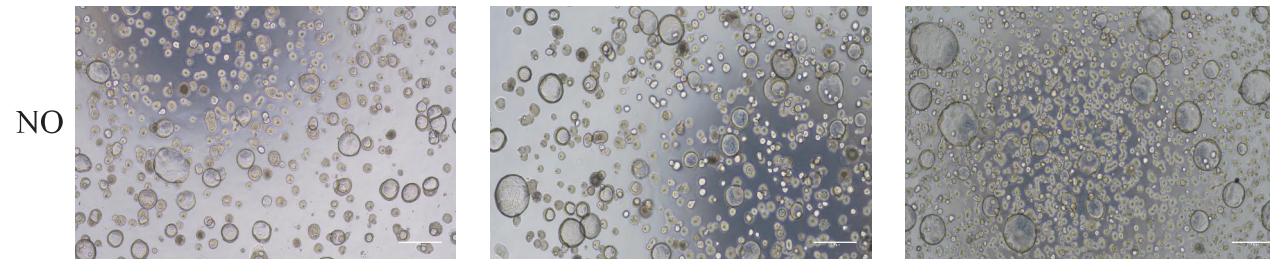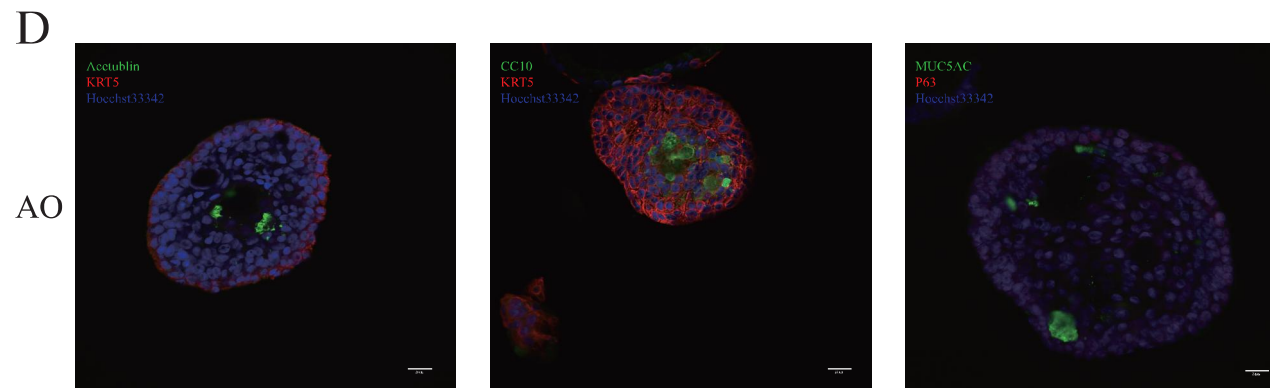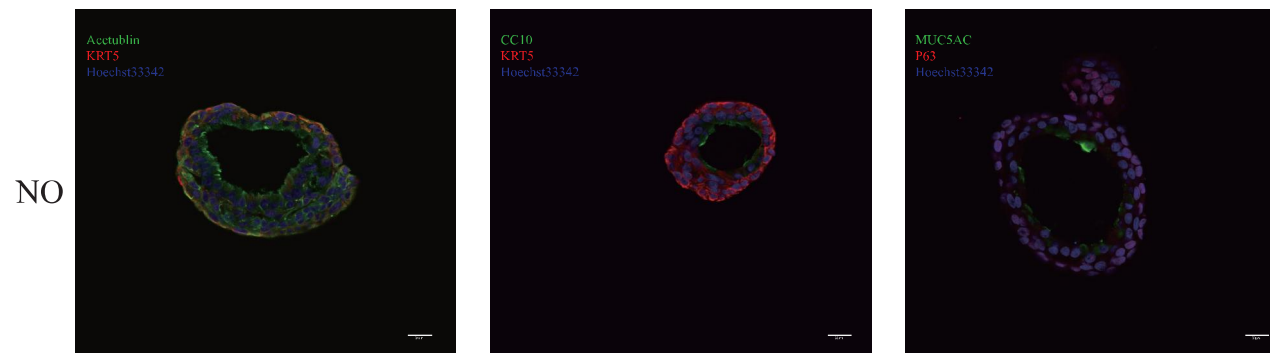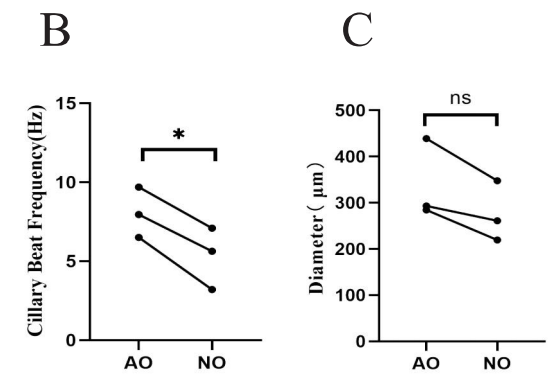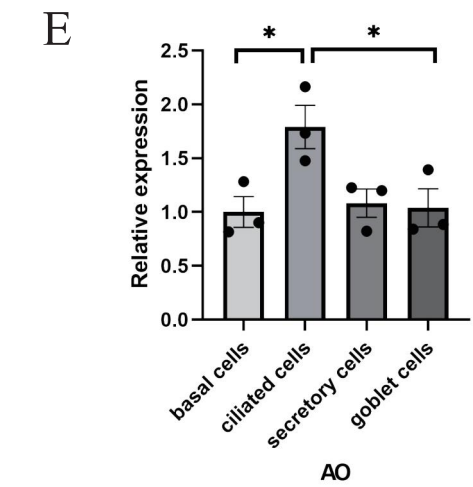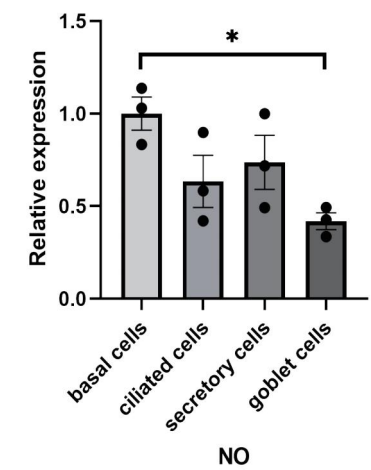

Supplement: Supplementary file 1 [file Image1.pdf]

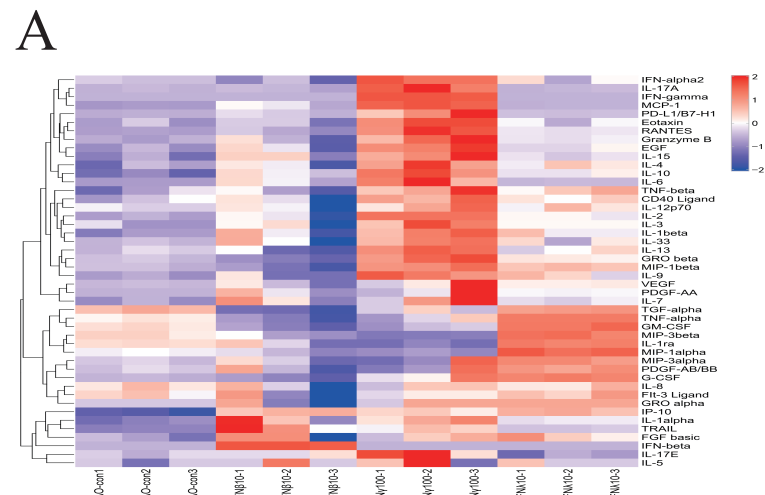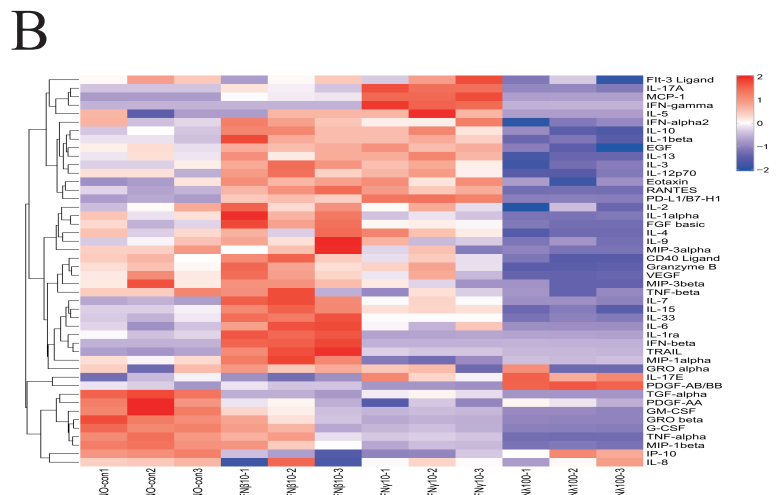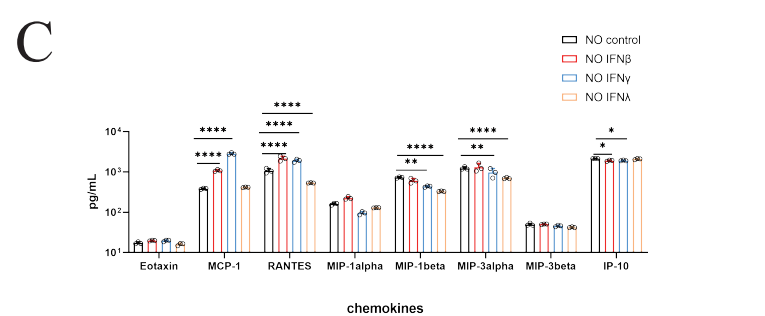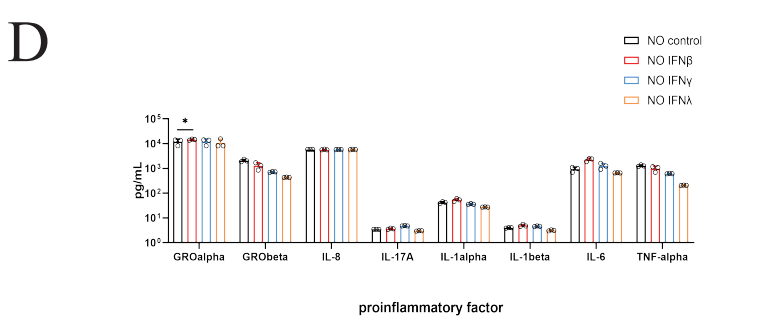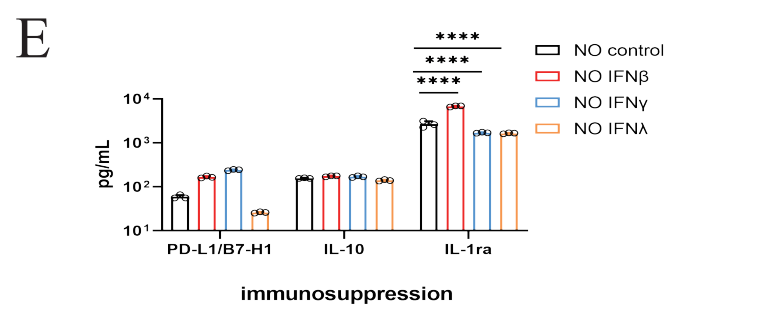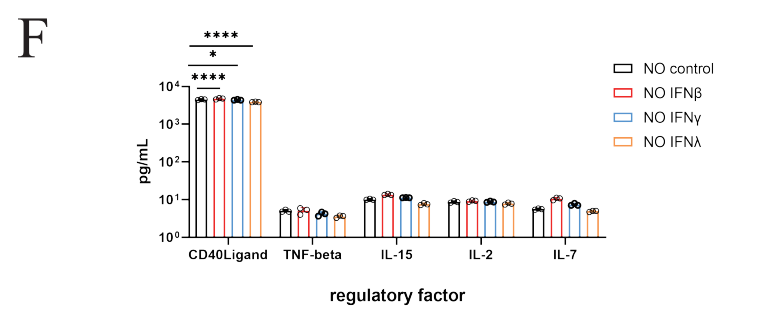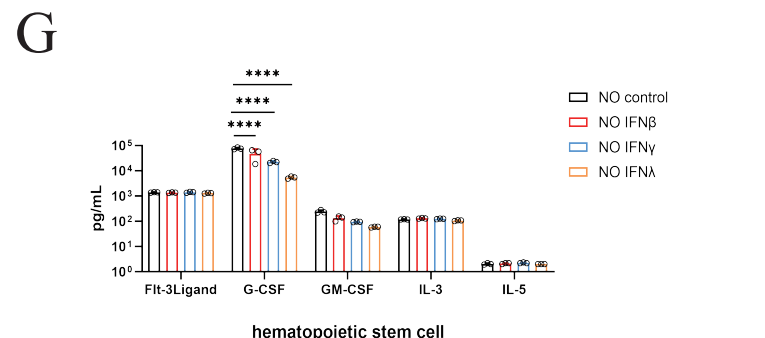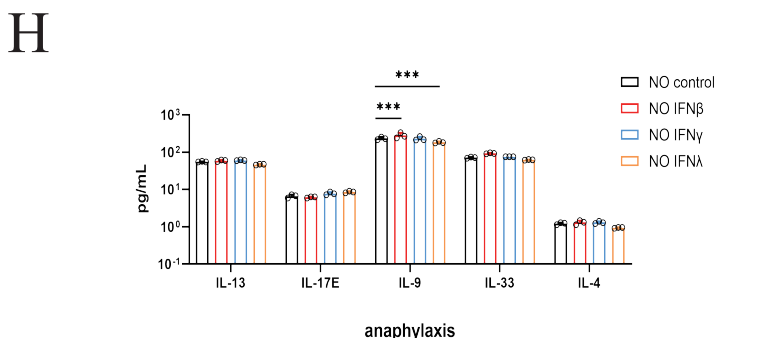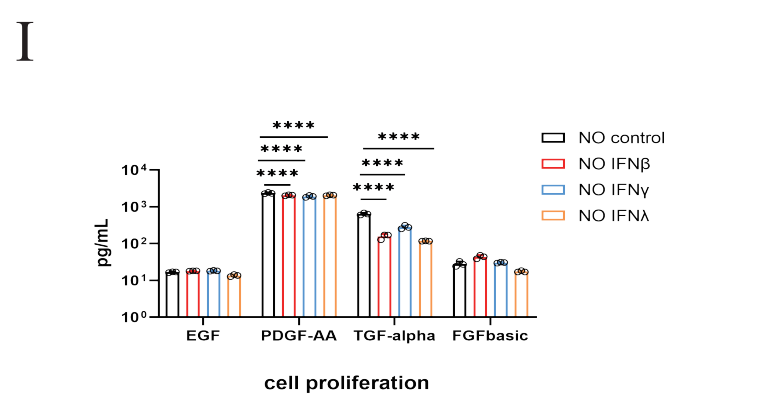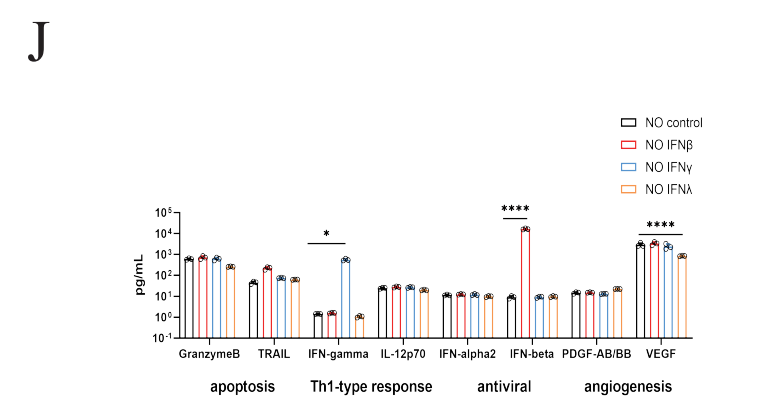

Supplement: Supplementary file 2 [file Image2.pdf]

A

AO

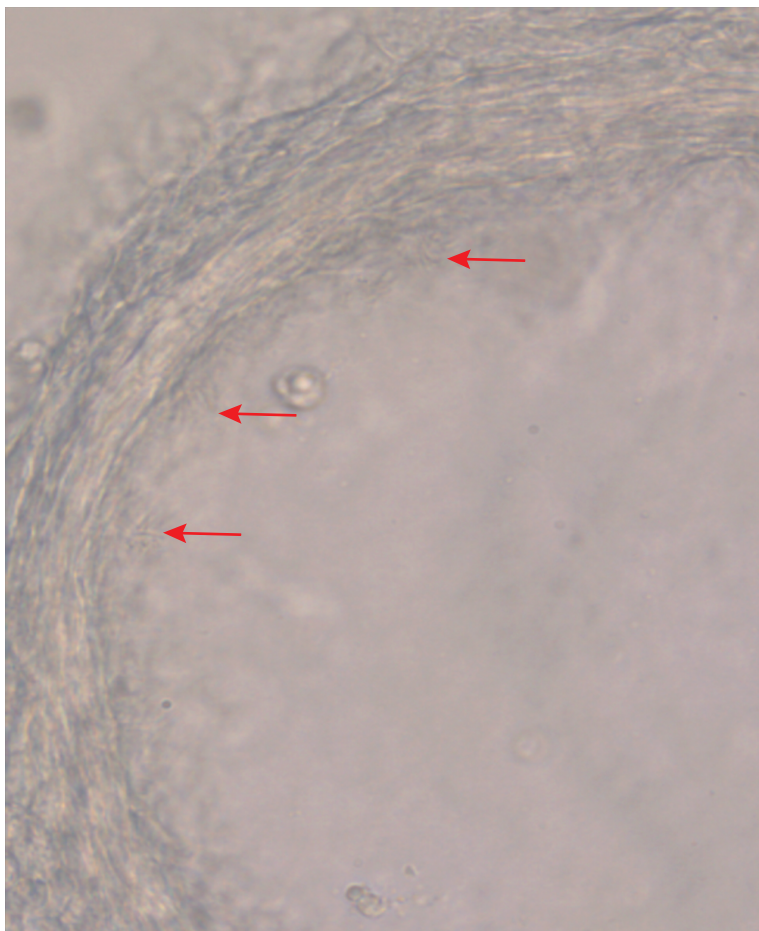

B

NO

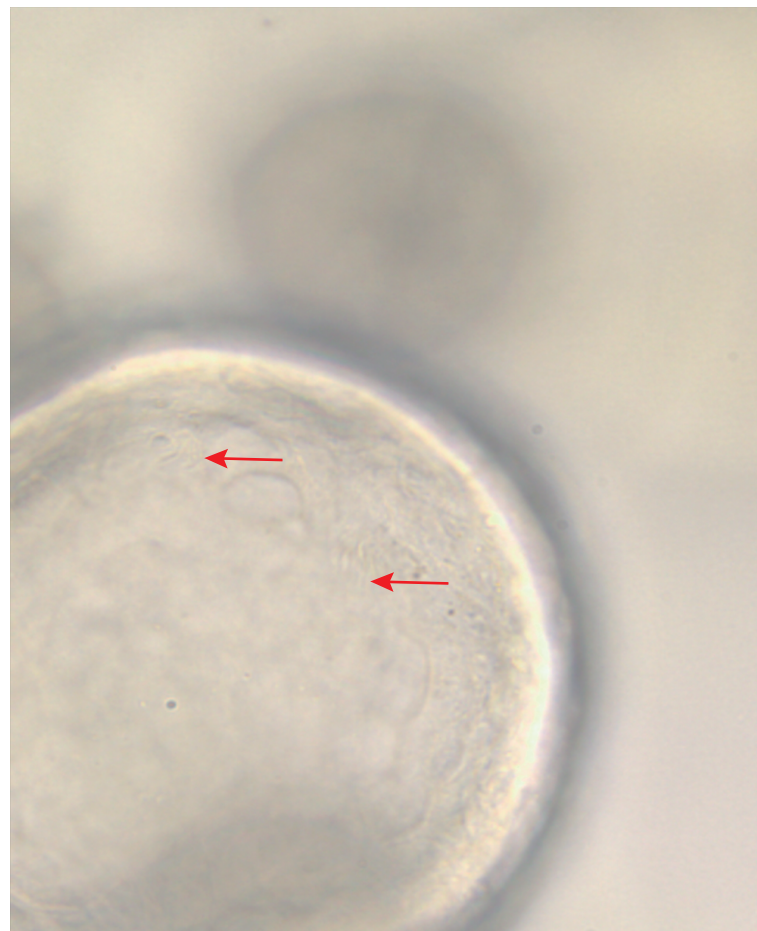

C

AO

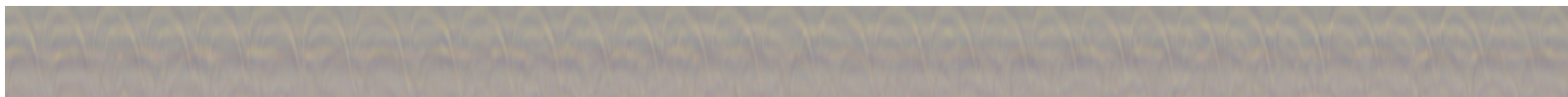

NO

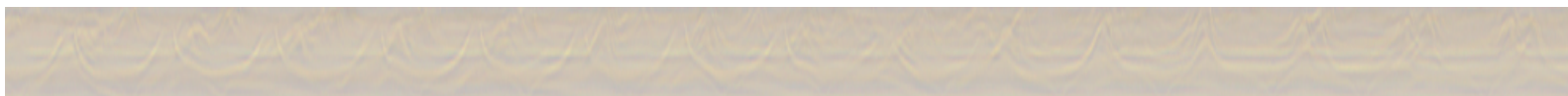

Supplement: Supplementary file 3 [file Image3.pdf]
