# Supplementary material for: Differential antiviral effects and immune responses in nasal and airway organoid during RSV infection: implications for interferon therapy
Source: Front Immunol. 2026 Feb 18;17:1754206. doi: 10.3389/fimmu.2026.1754206 (PMC12957070; doi:10.3389/fimmu.2026.1754206)
Supplement: Supplementary file 4 [file Table1.docx]

Supplementary Material

## 1.1 Graphical_Abstract


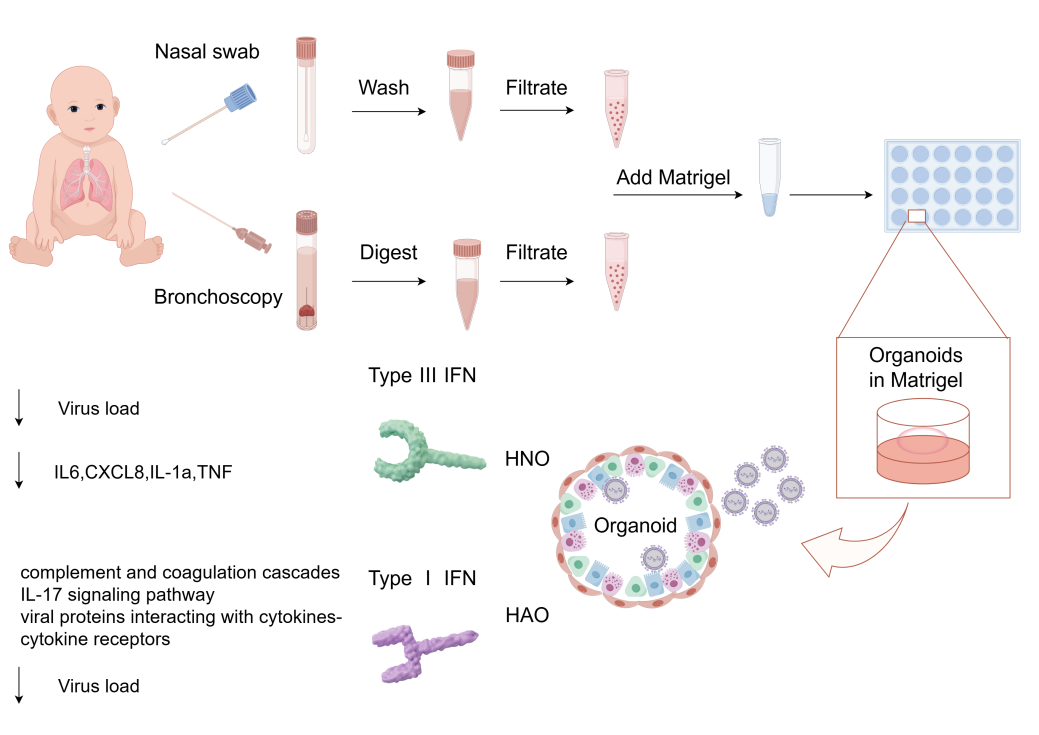


## Graphical_Abstract.This study created a special laboratory model that mimics a baby's respiratory system. Using this model, we discovered which antiviral treatments work best in different parts of the respiratory tract: interferon-lambda works best in the nose area, while interferon-beta is most effective in the airway region. Both treatments successfully combat the virus while keeping inflammation under control. These findings help doctors design more precise treatments for RSV infections in children, matching the right medicine to the right location in the airways.

## 1.2 Supplementary Figures

**Supplementar Figure 1.** Establishment of pediatric nasal and airway epithelial organoids. (A) Brightfield images of paired pediatric nasal and airway epithelial organoids at P3 passage, scale bar 500 μm. (B) Ciliary beating frequency in airway epithelium is higher than in nasal epithelium.(C) The diameters of paired nasal epithelial and airway epithelial organoids from the children showed no statistically significant difference.(D) Immunofluorescence staining of nasal and airway epithelial markers: ciliated cells (Acetublin), secretory cells (CC10), goblet cells (MUC5AC), and basal cells (KRT5/P63),scale bar 20μm. (E)Immunofluorescence quantification of airway organoids revealed a higher number of ciliated cells compared to basal and goblet cells.(F)Immunofluorescence quantification revealed a lower number of goblet cells compared to basal cells in nasal organoids. n = 3 biological replicates, each with 3 technical replicates.

**Supplementar Figure 2.** Cytokine Responses in Control and Interferon-Treated Groups During RSV Infection in Pediatric Nasal Epithelial Organoids. (A) Heatmap showing overall cytokine expression between control and interferon-treated groups during RSV infection in pediatric airway epithelial organoids. (B) Heatmap showing overall cytokine expression between control and interferon-treated groups during RSV infection in pediatric nasal epithelial organoids. Inflammatory factors were functionally categorized into 11 groups: chemotactic factors, pro-inflammatory factors, regulatory factors, anti-inflammatory factors, hematopoietic stem cell response, allergic response, cell proliferation, antiviral response, apoptosis,Th1 response and angiogenesis. Bar charts show differences in cytokine expression between the control and interferon-treated groups during RSV infection in pediatric nasalepithelial organoids. (C) Chemotactic factors. (D)pro-inflammatory factors. (E) anti-inflammatory factors. (F)regulatory factors. (G)hematopoietic stem cell response factors. (H)allergic response factors. (I)cell proliferation factors. (J)antiviral response, apoptosis,Th1 response and angiogenesis.* p < 0.05, ** p < 0.01, *** p < 0.001.

**Supplementar Figure 3.** Analysis of ciliary beating frequency in organoids. (A) Representative high-speed video frame of ciliary motion under 40× magnification (AO). Arrows indicate ciliary structures. (B) Representative high-speed video frame of ciliary motion under 40× magnification (NO). Arrows indicate ciliary structures. (C) The motion videos were converted into sinusoidal strip charts for waveform analysis, with frequency calculated based on pixel intervals over ten complete waveforms.

**Data Availability Statement**

The raw sequence data reported in this paper have been deposited in the Genome Sequence Archive (Genomics, Proteomics & Bioinformatics 2025) in National Genomics Data Center (Nucleic Acids Res 2025), China National Center for Bioinformation / Beijing Institute of Genomics, Chinese Academy of Sciences (GSA-Human: HRA014771) that are publicly accessible at [https://ngdc.cncb.ac.cn/gsa-human(1,2).](https://ngdc.cncb.ac.cn/gsa-human.)

1. Zhang S, Chen X, Jin E,Wang A, Chen T, Zhang X, et al. The GSA family in 2025: Q26

A broadened sharing platform for multi-omics and multimodal data. Genomics

Proteomics Bioinf. (2025) 23:qzaf072. doi: 10.1093/gpbjnl/qzaf072

2. CNCB-NGDC Members and Partners, Bao Y, Bai X, Bu C, Chen H, Chen H, et al.

Database resources of the national genomics data center, China national center for

bioinformation in 2025. Nucleic Acids Res. (2025) 53:D30–44. doi: 10.1093/nar/gkae978
